# Impact of HIV Status Notification on Risk Behaviors among Men Who Inject Drugs in Kermanshah, West of Iran 

**Published:** 2016-08-29

**Authors:** Alireza Noroozi, Ali Mirzazadeh, Ali Farhoudian, Ahmad Hajebi, Hamid Reza Khankeh, Peter Higgs, Hamid Sharifi, Bahram Armoon, Mehdi Noroozi

**Affiliations:** ^a^ Department of Neuroscience and Addiction Studies, School of Advanced Technologies in Medicine, Tehran University of Medical Sciences, Tehran, Iran; ^b^ Department of Epidemiology and Biostatics, University of California, San Francisco, CA, USA; ^c^ HIV/STI Surveillance Research Center, and WHO Collaborating Center for HIV Surveillance, Institute for Futures Studies in Health, Kerman University of Medical Sciences, Kerman, Iran; ^d^ Substance Abuse and Dependence Research Center, University of Social Welfare and Rehabilitation Sciences, Tehran, Iran; ^e^ Addiction and High Risk Behavior Research Center, Tehran Institute of Psychiatry, Faculty of Behavioral Sciences and Mental Health, Iran University of Medical Sciences (IUMS), Tehran, Iran; ^f^ Research Center in Emergency and Disaster Health, University of Social Welfare and Rehabilitation, Tehran, Iran; ^g^ Department of Clinical Sciences and Education, Karolinska Institute, Stockholm, Sweden; ^h^ Department of Public Health, School of Psychology and Public Health, La Trobe University, Melbourne, Australia; ^i^ Student’s Research Committee, School of Health, Shahid Beheshti University of Medical Sciences, Tehran, Iran

**Keywords:** Coarsened exact matching, People who inject drugs, Risk behaviors

## Abstract

**Background:** It is unclear whether knowing of current HIV status is associated with change in injecting behaviors among people who inject drugs (PWID) in Iran. The objective of the present study was to determine whether awareness of HIV positive status is associated with a reduction in injecting risk behaviors, after matching for socio-demographic characteristics.

**Methods:** Five hundred male PWID were recruited in 2014 from two drop-in centers (DICs) in Kermanshah west of Iran. Trained interviewers collected data on socio-demographic characteristics, HIV testing and drug-related risk behaviors over the last month prior to interview using a structured questionnaire. Our primary exposure of interest was awareness of HIV status used to group participants into three categories: positive, negative, unaware. We used coarsened exact matching to make the three groups statistically equivalent based on age, place of residence, education and income, and then compared them regarding the proportion of borrowing, lending and reuse of syringes.

**Results:** Matched sample (n=320) had a mean age ± standard deviation (SD) of 33.5 ±7.6 yr. Overall, 25% (95% CI: 14%, 32%) of participants reported "borrowing a syringe" in the past month and 15% (95% CI: 7%, 22%) of them reported "lending a used syringe" to others in the past month. In comparison to PWID who were unaware of their HIV status, those knew they were HIV positive (OR 1.68, CI95%1.32–2.81) or negative (OR 1.54; 95% CI: 1.28, 2.71) were both more likely to report borrowing syringes in past month.

**Conclusions:** PWID WHO know they are positive for HIV are more likely to borrow another person’s syringe, to report reuse of their own used syringes and less likely to report lending their syringes to others. Strategies to scale up HIV testing and counseling for PWID, which also increase awareness of HIV status, may decrease injecting related the risk behaviors.

## Introduction

Drug injection is responsible for a large proportion of the blood-borne transmitted disease burden in both developed and developing countries^1^. Behaviors associated with injection drug use that are responsible for HIV transmission, such as sharing of injection equipment (especially needles and syringes), and unprotected vaginal and anal intercourse with an injecting drug user are considered as main routes of HIV and other blood-borne viruses (NNV) transmission^2-4^. It is estimated that between 170,000 and 230,000 people use drugs through injection^5,6^ and about two-thirds of new HIV infections are attributed to unsafe drug injection in Iran^5-8^. The prevalence of hepatitis C virus (HCV) infection is also very high amongst people who inject drugs (PWID) in the country, with studies reporting 50% to 75% of them are HCV positive depending on the setting of the study^9^.

Approaches to HIV and HCV risk reduction among PWID include drug treatment, HIV testing and counseling programs, street-based outreach conducted by peer educators, individual and group counseling, community-level interventions to change injecting norms, and needle syringe programs (NSPs) to provide sterile injection equipment^10-13^. In Iran, NSPs are well established having been developed and implemented since 2002 ^14, 15^. Although such programs contribute to stabilizing the HIV epidemic among PWID in Iran, unsafe injection and particularly needle/syringe sharing (12.6%) are still reported by PWID^9,16,17^.

Screening PWID for HIV and HCV infection and providing risk reduction counseling have been recommended by WHO to reduce BBVs transmission^18^. Evidence supporting effectiveness of HIV and HCV screening and counseling on change in injecting high-risk behaviors among PWID are weak^19^. BBVs screening and counseling programs among PWID receiving opioid maintenance treatment were moderately cost-effective as compared to no screening. It is also associated with 5% decrease in needle-sharing which improve its cost-effectiveness^20^.

Few studies internationally have examined the impact of HIV diagnosis on injection risk behaviors^19^. Most studies on HIV and HCV screening and awareness have relied on cross-sectional study designs and yielded diverse results^21^. PWID, diagnosed with HIV or HCV, and receive their test result, will subsequently reduce their injecting risk behavior in order to decrease risk of transmitting HIV and HCV to others^22^. There is no change in injecting drug use behaviors including syringe and other equipment sharing amongst young PWID diagnosed with HCV infection^23^. An increase in the use of new needles and syringes in the three months following HCV testing and counseling among PWID in Melbourne is reported, but no difference was found between people received positive or negative HCV results^24^. Hence, it is unclear whether knowing of one’s HIV status influences injecting behaviors and reduces the risk of BBV transmission.

Understanding the fact, that awareness of current HIV status has effect on different behavioral outcomes for PWID is important for policymaking and planning purposes. Our study was an observational study using Coarsened Exact Matching (CEM) to match participants who were aware of their HIV status (positive and negative) with those who were unaware of their current HIV status in order to ensure statistically equivalent comparison groups to estimate the effect HIV status has on injection risk behavior^15^.

The objectives of the present study were to determine whether PWID knowledge of current BBVs status was associated with a reduction in injecting behavior compared to PWID who do not know their current HIV status. The HIV epidemic in Iran was first observed in Kermanshah in 1996 where the most affected population was PWID. Since then many harm reduction services have been implemented in the city, including the needle and syringe programs (NSPs) and opioid maintenance treatment (OMT) programs. HIV remains highly prevalent among PWID in Kermanshah and The National AIDS Control Program in Iran conduct HIV surveillance for monitoring HIV trends^13^ and Kermanshah is a sentinel site, we chose it for our study site. This study can provide valuable insights into the effectiveness of current BBV counseling and testing programs and help to provide practical training in the implementation and development of harm-reduction strategies.

## Methods

We used data from a cross-sectional survey using snowball sampling to recruit 500 PWID from September to December 2014, in Kermanshah, west of Iran. To be eligible for the study, participants were required to be over 18 years old, to inject illicit drugs at least once in the past month which was verified by presence of "stigmata," or track marks, to be able to speak and comprehend Farsi enough to respond to survey questions and to provide informed consent. Exclusion criteria were history of methamphetamine use in the post month and absence of track marks. All respondents who meet the screening criteria were asked to participate in the study. During data collection, 500 individuals were screened. Among them, 10 individuals were not eligible for participation, and 10 individuals refused.

To gather study data, a structured questionnaire was used by trained interviewers. The questionnaire included modules on socio-demographic characteristics, sexual behaviors, HIV testing, participation in harm reduction program activities, frequency of injection and drug-related risk behaviors such as sharing (borrowing or lending) of syringes/needles and other paraphernalia (i.e., cookers, cotton filters, and rinse water), reuse of syringes and number of injecting partners they have shared syringes/needles with over the past month prior to the interview. We validated the content of our questionnaire with inputs from eight behavioral sciences, epidemiology and harm reduction experts. The study protocol and all the procedures were reviewed and approved by the Research Ethics Committee of the Kerman University of Medical Sciences (KUMS), of approval number: k/93/204.

We assessed six outcomes of interest, all treated as binary variables including not lending needles or syringes in the past month, not borrowing needles or syringes in the past month, reuse of own syringes, not sharing paraphernalia in the past month, and consistent condom use with any type of sexual partner in the past month. The exposure of interest was known HIV status, defined by self-report of current HIV status. Each variable was coded as a dichotomous measure, indicating that some respondents were unaware of their current HIV status. We also considered several other measures. Socio-demographic variables include age, education, occupation and monthly income. Sexual behaviors include total number of sexual partners in the past year, type of sexual partners (commercial, causal, regular), and whether the respondent reported having sex while intoxicated by drugs/alcohol in the past month.

Coarsened Exact Matching (CEM) is a statistical matching technique used to improve causal inferences of observational studies. It is recommended when an experimental design is not feasible. Here CEM was applied to match PWID known to be HIV positive and HIV negative with those PWID who did not know their current HIV status. Covariates were chosen in order to ensure statistically equivalent comparison groups to estimate the effect of HIV status notification on injection risk behaviors. CEM assigns each case into one of a specified set of strata in which members are exactly matched on a set of coarsened, i.e. categorized, variables^25^. Matched members are then assigned a weight specific to that stratum and representative of the proportion of all members present in the stratum^26^. Based on the six -selected matching variables, we calculated a statistical measure called L1 distance. It varies between 0 and 1 and any values close to zero mean that the matching is perfect and ensures the comparability of the two groups^27^. We calculated the L1 before and after applying CEM, and we observed that L1 decreased from 0.37 to 0.00004 after coarsened exact matching indicating minimal imbalance between the two groups we wanted to compare. We then reported the descriptive statistics for the pooled sample and matched sub-sample. We applied a logistic regression to estimate the effect of knowledge of current HIV status on injection high-risk behaviors. The effects were reported as OR and 95%CI. For all analyses *P*<0.05 was considered significant. All analyses were undertaken in Stata version 12 (Stata Corp, College Station, Texas)

## Results

A total of 480 male PWID participated in this study. The characteristics of participants in pooled (unmatched) and matched samples are presented in Table 1. The matched sample (n = 320) had a mean age ± standard deviation (SD) of 33.5 ± 7.6(IQR, 25.6–42.4) yr. The mean and median durations of injection drug use were 6.0 ± 4.6 and 3.2 (IQR, 3.6–11.1) yr. The majority of respondents were single (73%), 53% lived with their families, and 90% had a monthly income of less than $150 (the average family monthly income was $470). While 40% of study subjects did not know their HCV status, 32% reported being HCV positive. Regarding HIV status, 22% did not know their current sero-status and 14% reported themselves as HIV positive.

**Table 1:** Population characteristics and risk profile for people who inject drugs in matched and unmatched samples of PWID, Kermanshah, west of Iran 2014

**Variables**	**Pooled sample,** **n (%) (n=480)**	**Matched sample,** **n (%) (n=320)**
Age (yr)		
<30	216 (44.3)	166 (52.4)
30-39	144 (30.2)	90 (28.2)
≥40	120 (25.5)	63 (20.4)
Education		
Primary or below	120 (24.2)	64 (20.4)
High school	192 (40.3)	160 (50.3)
Diploma and high	168 (35.5)	96 (39.3)
Marital		
Single	336 (70.3)	233 (73.5)
Married	48 (10.2)	26 (8.2)
Divorce	72 (14.2)	38 (11.2)
Widowed	24 (5.3)	23 (7.1)
Monthly income		
less than 150 US$	400 (83.3)	290 (90.4)
150 US$ or more	80 (16.7)	40 (9.6)
Age of first injecting drug use		
<25	270 (61.4)	224 (70.1)
25-30	100 (21.3)	64 (20.2)
30 and upper	87 (17.3)	32 (9.7)
Age of first drug use (yr)		
<25	417 (87.8)	280 (88.3)
25-30	51 (9.1)	30 (7.6)
≥30	12 (3.1)	10 (2.1)
Aware of HCV Status	303 (63.2)	193 (60.3)
Aware of HIV Status	402 (83.7)	281 (87.8)
Borrowing a syringe in past month	135 (28.2)	80 (25.1)
Lending a used syringe in past month	77 (16.2)	48 (15.3)
Borrowing or lending paraphernalia in past month	283 (58.9)	176 (55.3)
Reuse of own syringes in past month	355 (73.9)	227 (70.9)
Mean number of injecting partners (SD)	2.43 (1.4)	2.2 (1.3)

The mean and standard deviation age of age at first illicit drug use were 22.4 ± 5.6 and (IQR, 20.6–26.1), respectively. About 25% of matched respondents reported “borrowing a syringe” in the past month. Similarly, 15% of matched participants reported “lending a used syringe” to other people within the last month. The recent borrowing or lending of injecting paraphernalia was reported by 55% of participants. In addition, 71% of matched participants reported “reuse of their own syringes” in the month prior to the interview. The mean number of injecting partners whom the study participants had shared syringe/needle or paraphernalia with was 2.28±1.35 (Table 1).

We present the effect of knowing current HIV status, on different injection risk behaviors in the pooled and matched samples in Table 2. In the matched sample, PWID who reported being HIV positive were more likely to report borrowing syringes in past month as compared to PWID who did not know their HIV status and PWID who reported being HIV negative status (OR: 1.7 95% CI:1.32, 2.81 and OR: 1.54, 95% CI:1.28, 2.71). PWID who knew their HIV positive status reported less frequent syringe lending as compared to PWID who did not know their HIV status (OR: 0.22, CI95%: 0.1, 0.84, P=0.022) and PWID who report HIV negative status (OR: 0.25, CI: 95% 0.74, 2.17).

**Table 2:** Logistic regression estimates of known HIV status and safer injecting practices in matched and unmatched samples of PWID, Kermanshah, west of Iran 2014

**Behavioral outcomes**	**Pooled sample OR (95% CI)**	**Match sample** **OR (95% CI)** ^a^
Borrowing a syringe in past month		
PWID unaware of HIV status	1.00	1.00
Aware HIV+ status	1.71 (1.30, 1.86)	1.68 (1.32, 2.81)
Aware HIV- status	0.51 (1.23, 1.77)	1.54 (1.28, 2.71)
Lending a syringe in past month		
PWID unaware of HIV status	1.00	1.00
Aware HIV+ status	0.25 (0.18, 0.77)	0.22 (0.10, 0.84)
Aware HIV- status	0.34 (0.18, 0.64)	0.31 (0.15, 0.60)
Sharing a paraphernalia in past month		
PWID unaware of HIV status	1.00	1.00
Aware HIV+ status	0.7 (0.42, 1.75)	0.68 (0.30, 2.32)
Aware HIV- status	0.89 (0.43, 2.04)	0.78 (0.47, 3.22)
Syringe reuse in past month		
PWID don’t Know HIV status	1.00	1.00
To know HIV+ status	2.31(1.19,4.51)	2.38 (1.60, 4.68)
To know HIV- status	3.44 (1.27,5.72)	3.54 (1.30, 5.42)
Consistent condom use with any type of sexual partner		
PWID unaware of HIV status	1.00	1.00
Aware HIV+ status	3.34 (1.70, 5.20)	3.22 (1.50, 4.80)
Aware HIV- status	3.24 (1.90, 4.86)	3.19 (1.50, 4.60)

^a^The matched subsample was made by considering age, income, education, access to NSP facilities and city of residence.

Participants reported reuse of their own syringe more frequently, if they knew they were HIV positive (OR: 2.38, 95% CI: 1.6, 4.68, P=0.031) as compared to PWID who did not know their current HIV status or reported HIV negative status. Reporting being HIV positive or negative increased the chance of syringe reuse by 3.54 and 2.38, respectively. Recent paraphernalia sharing was reported less among PWID who were known to be HIV positive (OR: 0.86, 95%CI: 0.30, 2.32, P=0.311) or negative as compared to PWID who did not know their current HIV status (OR: 0.78, 95% CI: 0.47, 3.38, P=0.231). However, neither was statistically significant. There was a statistically significant relationship between awareness of current HIV status with consistent condom use with all sexual partners (regular, casual or paid) (OR: 3.22 95% CI: 1.5, 4.8).

## Discussion

Using the matched analysis, we found that reporting being HIV positive was associated with more borrowing and reuse of own used syringes and less lending syringes. Recent researches from both Australia and Canada have suggested there were protective behavioral changes for PWID associated with the current BBVs notification process^22,24^. The concept of “teachable moment” has been introduced, to describe health conditions, which motivate individuals to adopt risk-reduction health behaviors^19^. We observed a significant effect of HIV positive status notification on needle and syringe borrowing. This finding is in line with evidence from France, where HIV-positive PWIDs who were aware of their sero-status were more likely to borrow injection paraphernalia^28^. In Baltimore, PWID who reported awareness of their HCV status were more likely to share as compared with those who were unaware of their status^22^. Qualitative evidence from the United Kingdom, suggests that PWID infected with HIV and HCV were less likely to be concerned about borrowing injecting equipment from others, based on the assumption that they were already infected^29^. For PWID who know their HIV positive status, this may be a cue prompting motivation for change to improve their own health^19^. These findings emphasis the importance of providing education and counseling^30^ on ongoing risk posed by other BBVs for HIV positive PWID in harm reduction programs.

Similarly to France, where HCV positive PWID who were aware of their status were less likely to lend syringes to others^30^, PWID in our study who reported currently being HIV positive were more likely to decrease their frequency of lending their own used needles and syringes. This is likely due to their conscious desire to prevent the BBVs transmission to others. In Montréal, Canada participants reported a decrease in syringe sharing as well as cocaine and heroin injection following notification of their BBVs results whether positive or negative^19^. Together with the findings from our study, these suggest a need to strengthen current HIV and HCV testing programs. Expanding current counseling and testing programs, developing targeted testing for PWID, and providing diverse platforms to share HIV and HCV risk reduction information will add considerable value to current prevention efforts.

In this analysis, we found a statistically significant relationship between the reuse of PWID own syringes and awareness of their HIV status. Our findings show indirect sharing of other injection paraphernalia (i.e., cookers, cotton filters, and rinse water) was reportedly less among PWID who know their current HIV status; however, these were not statistically significant. Previous studies have shown no change in injecting equipment borrowing following a diagnosis of HCV infection amongst young PWID in US^22,24^. This may be due to lack of young PWID’s knowledge regarding risk of BBVs’ transition through sharing paraphernalia. Recent studies have highlighted the risk of BBVs with suggestions that one-half of the new HCV infections observed among PWID can be accounted for through indirect sharing of injection equipment (i.e., cookers and cotton and ﬁlters) ^18,30-32^. Our finding that more than half the PWID who knew they were HIV positive continued to report the sharing of injection equipment highlights the need for targeted behavioral interventions to reduce these high-risk behaviors. In particular, PWID in our study appear to be unaware of the risks of BBVs transmission associated with indirect sharing of injection equipment.

This study is a post hoc analysis of data from a study designed to investigate risk-behaviors for HIV infection among PWID in Iran. This was not a study to assess the impact of HIV counseling and testing on risk behaviors. However, our findings provide valuable insight into the effect of HIV status awareness on injecting risk behaviors.

There are limitations to our study. Like any observational study, we can only report the association of exposure with high-risk behaviors. Furthermore, our data were based on participants self-report, therefore may be subject to recall and/or social desirability bias^33^. We might also misclassify PWID based on knowing their HIV status as it was based on their self-report. This may lead to attenuating the true differences in the effect of the two groups. We matched the three groups based on age, income, education, access to NSP facilities and city residence; however, we could not account for other factors such, access to methadone maintenance therapy.

## Conclusions

A large reduction in risky behaviors was observed among PWID who were aware of their current HIV status. HIV status notification, whether results are positive or negative, could enhance the impact of counseling and the willingness of PWID to initiate injecting related changes in behaviors. Our study highlights the need to link regular HIV counseling and testing with drug treatment and harm reduction programs for all PWID.

## Acknowledgments

We gratefully thank all staff in the drop-in centers (DICs) in Kermanshah who contributed in recruiting (Ms. Barkhordar) and data collection/interview (Mr. Azad and Mr. Amini). We thank participants for their time and interest in the study. We also want to appreciate Mostafa Shokohi from the Regional Knowledge Hub for HIV Surveillance who provided valuable input to the study protocol and the questionnaire.

Authors’ contributions: Study concept and design: HS and AM. Analysis and interpretation of data: MN and SH. Drafting the manuscript: MN, AM and AN. Critical revision of the manuscript: AN, PH, HS, SH, AH and AM.

This research funded by Substance Abuse Prevention and Treatment Office (SAPTO) of Mental Health, Social Health and Addiction Department (MeHSHAD) of Ministry of Health and Medical Education, I.R. of Iran.

## Conflict of interest statement

All other authors have no conflicts of interest to be declared.

## Highlights

- We found that reporting being HIV positive was associated with more borrowing and reuse of own used syringes and less lending syringes.
- PWID who reported currently being HIV positive were more likely to decrease their frequency of lending their own used needles and syringes.
- Strategies to scale up HIV testing for PWID which also increase awareness of HIV status may decrease injecting related the risk behaviors.
